# The Impact of Immunomodulatory Components Used in Clinical Nutrition—A Narrative Review

**DOI:** 10.3390/nu17050752

**Published:** 2025-02-21

**Authors:** Aleksandra Raczyńska, Teresa Leszczyńska, Piotr Skotnicki, Aneta Koronowicz

**Affiliations:** 1 Department of Human Nutrition and Dietetics, Faculty of Food Technology, University of Agriculture in Krakow, al. Mickiewicza 21, 31-120 Krakow, Poland; aleksandra.raczynska_wtz@student.urk.edu.pl (A.R.); teresa.leszczynska@urk.edu.pl (T.L.); 2 Department of Surgery with a Sub-Department of Oncological Surgery, Independent Public Health Care Facility in Bochnia “District Hospital” Named after Blessed Marta Wiecka, ul. Krakowska 31, 32-700 Bochnia, Poland; pskotnicki@vp.pl

**Keywords:** malnutrition, clinical nutrition, immunomodulating ingredients

## Abstract

Background: Malnutrition is a clinical condition that leads to unfavourable changes in health. It affects 35–55% of hospitalized patients, and in the case of cancer, this prevalence rises to 40–90% of patients. Screening nutritional status is essential for preventing undernutrition, which is crucial as its treatment. Undernutrition in patients after severe injuries significantly increases catabolic changes. Cytokines and hormones, such as epinephrine, glucagon, and cortisol, are released, which can increase energy expenditure by 50%. Properly conducted nutritional treatment aims to maintain or improve the nutritional status of patients whose nutrition with a natural diet is insufficient, moreover, in some cases, treatment of the underlying disease. Methods: This study is a narrative review focused on immunonutrition. The search for source articles, mainly from the last 10 years, was conducted in the PubMed and Google Schoolar databases, as well as in printed books. The key words used were “malnutrition”, “inflammation”, “clinical nutrition”, “immunomodulatory components”, “nutritional status assessment”, “enteral nutrition”, “parenteral nutrition”, and their combinations. Results: Providing substances such as omega-3 fatty acids, glutamine, arginine, nucleotides, antioxidants, and prebiotic fiber has a beneficial impact on immunological and anti-inflammatory pathways. The above-mentioned ingredients may inhibit the secretion of pro-inflammatory cytokines, activate anti-inflammatory cytokines, stimulate immune cells, and have a beneficial effect in allergic diseases, respiratory infections, or wound healing. Conslusion: Immunonutrition can be administrated via oral, enteral, and parenteral routes. It is crucial to highlight the importance of proper nutritional status in patients. The relationship between inflammation and malnutrition creates a vicious cycle, where one negatively affects the other due to increased metabolic demand, loss of appetite, weakened immune system, and gut dysbiosis.

## 1. Introduction

Proper nutrition and adequate nutritional status are key areas of today’s treatment. Malnutrition affects up to 55% of hospitalized patients [1]. Often unnoticed, left without intervention due to insufficient staff awareness, it brings further negative effects and deterioration of the patient’s clinical condition [2]. A significant correlation has been proven between the patient’s nutritional status and the effects of treatment, reduction of complications, and length of hospital stay. Despite this, malnutrition is still often underestimated. In the case of malnutrition, patients should be approached holistically. The whole includes the physical but also the mental health of the sick. In such a condition, cognitive disorders and depression often occur, which intensifies the slowdown of treatment. Untreated malnutrition leads to systemic disorders of the body, i.e., heart, kidney, and respiratory failure, and may ultimately lead to multi-organ failure. It concerns patients in many hospital wards, such as oncology, surgery, internal medicine, gynecology, and orthopedic departments. The model for proper management and limiting the development of malnutrition is the screening assessment of nutritional status and the introduction of appropriate nutritional intervention [1,2].

Clinical nutrition includes oral nutrition by supplementing the natural diet with food for special medical purposes, enteral nutrition, and parenteral nutrition. The introduction of a regimen appropriate to the patient’s clinical condition and needs allows for the improvement of anthropometric and laboratory parameters [3].

One of the important phenomenona accompanying hospitalized patients is persistent inflammation as a response of a body to injury. The influence of selected substances, such as omega-3 fatty acids, glutamine, and nucleotides, on the secretion of inflammatory mediators, such as cytokines, chemokines, or interferons, has been proven [4]. An important factor is maintaining a balance in the functioning of the immune system. Excessive levels of secreted cytokines can affect tissue damage, contributing to neurodegenerative and autoimmune diseases [5]. This narrative review attempts to indicate the links between the proper nutritional status of hospitalized patients and the inflammatory state accompanying the disease. Nutritional intervention, appropriately selected and carried out and, in selected cases, enriched with components affecting the immune system, is the key. The authors attempt to indicate the role of such nutrition in various disease entities and patient groups while discussing some details and principles, including the assessment of nutritional status, enteral and parenteral nutrition, and basic mechanisms of inflammation.

Our narrative review can contribute to important recommendations in the field of immunonutrition, such as (1) establishing universal and standardized screening tools in hospitals and care facilities to detect malnutrition earlier, ensuring appropriate nutritional support is introduced before the condition worsens; (2) developing patient-specific nutritional strategies that factor in individual needs, comorbidities, and disease states to optimize treatment outcomes (e.g., adapting the proportion of macronutrients or adding immunomodulating supplements like omega-3 fatty acids, glutamine, or vitamin D); and (3) broadening the use of immunomodulating nutritional ingredients, especially in high-risk patients (e.g., those with cancer, severe injuries, or autoimmune conditions). This could include routine supplementation of omega-3s, arginine, and nucleotides.

Methods: The search for source articles was conducted in the PubMed and Google Schoolar databases, as well as in printed books. The search was conducted between May and November 2024. The key words used were “malnutrition”, “inflammation”, “clinical nutrition”, “immunomodulatory components”, “nutritional status assessment”, “enteral nutrition”, “parenteral nutrition”, and their combinations. An effort was made to select articles from reputable journals, in English and Polish, not older than 10 years, unless these are key data for the review, such as NRS 2002 or the SGA scale.

## 2. Malnutrition

According to the World Health Organization (WHO), American Society for Parenteral and Enteral Nutrition (ASPEN), and European Society for Clinical Nutrition and Metabolism (ESPEN) guidelines, malnutrition is defined as an imbalance in nutrient intake or impaired processes of nutrient utilization. It includes clinical conditions such as undernutrition, but also overnutrition, i.e., overweight and obesity, and micronutrient deficiencies, as well as diet-related noncommunicable diseases [6,7,8]. As it is commonly known, the quality of nutrition, its quantity, and its individual components affect the condition of a body and have a preventive or modulating effect on selected diseases. A particular impact will also concern the ageing processes and physical capacity. Malnutrition is a disease that leads to unfavourable changes, including alterations in body composition, physical and mental condition, and an element predicting a worse course of the underlying disease [2].

There are many causes of malnutrition, but most often, it is the result of accumulation of several of them. According to the article by Szczygieł et al., it affects 35–55% of hospitalized patients, and in the case of cancer, this prevalence rises to 40–90% of patients. Insufficient intake of nutrients, increased demand during illness, or improper digestion and absorption processes are just some of the reasons, and their aetiology is much more complex [1]. One of the key factors is inflammation associated with some diseases [9]. The nutritional status of patients with brain injury was examined in the first week of hospital stay and then in the third week. The conclusions drawn indicate an increase in malnutrition from 45% to 76%, which also draws attention to the problem of hospital malnutrition [10].

Elderly patients are a group particularly vulnerable to the development of malnutrition, especially undernutrition. This population often includes individuals with multiple comorbidities, chronic inflammation, prolonged hospital stays, and physiological changes occurring at the level of hormones and enzymes [11]. Depression, low mood, suffering after the loss of relatives, and also bad economic conditions are problems that seniors struggle with on a daily basis, significantly affecting the deterioration of appetite and the general condition of the patient. Moreover, lack of teeth, problems with dentures, or dysphagia create another physical barrier for them [12]. Various causes of decreased appetite are suspected, not only a decrease in energy expenditure but also an increased feeling of satiety [13].

### 2.1. Types of Malnutrition

There are different types of malnutrition, such as kwashiorkor, marasmus, and mixed. According to ESPEN, among malnutrition, it is necessary to distinguish disease-related malnutrition (DRM) with inflammation (divided into chronic DRM with inflammation and acute disease- or injury-related malnutrition), DRM without inflammation (synonym: on-cachectic DRM), and malnutrition/undernutrition without disease (synonym: non-DRM divided into hunger-related malnutrition and socioeconomic or psychologic related malnutrition) [14].

(a) Kwashiorkor is characterized by abdominal swelling (so-called ascites) caused by disturbances in the acid-base and water-electrolyte balance. This may be related to the decreasing amount of proteins in the blood serum, mainly albumins. These molecules affect the oncotic pressure, leading to water retention in the body. This also correlates with the increase in the ADH hormone and renin, which causes an increase in swelling [15]. However, this is not the only cause of swelling; other factors are also necessary. Researchers point to the influence of the lymphatic system [16].

(b) Marasmus, otherwise known as protein-energy malnutrition, develops slowly; it is manifested by a general decrease in food intake, which is often associated with the occurrence of infectious diseases, especially respiratory and gastrointestinal diseases. The first effect of the lack of nutrients is stunted growth (in children), followed by weight loss. This condition is characterized by loss of adipose tissue, reduced muscle mass, lack of oedema, and significant changes in visceral proteins. The concentration of albumin and plasma proteins is usually normal or slightly changed despite a weight loss of 40% or more [17].

(c) The mixed type is often diagnosed, especially in chronically ill patients [18]. This type of malnutrition combines the clinical features of marasmus and kwashiorkor. It is characterized by the presence of oedema (with or without skin lesions), muscle atrophy, and a decrease in subcutaneous fat tissue, as in the case of marasmus. During the nutritional process, the oedema subsides, and the patients acquire clinical symptoms of marasmus [17].

Cachexia (synonym: DRM with inflammation), caused by a chronic disease (e.g., cancer), is also considered malnutrition. It is divided into the following stages:

- During precachexia, there is a loss of body weight less than or equal to 5% over a period of 6 months, chronic or temporary but recurrent inflammation (CRP > 5.0 mg/L), and symptoms such as loss of appetite and gastrointestinal problems, e.g., nausea or vomiting.
- A patient with cachexia has a weight loss of more than 5% or a BMI below 20 and a weight loss of more than 2% and three out of five other indicators, such as fatigue, reduced muscle strength or mass, abnormal biochemical parameters, or anorexia [14].

The prognosis for survival is about 3 months. The triad of cachexia disorders is: continuous weight loss (concerning, in particular, lean body mass), resistance to nutritional treatment, and exacerbation of symptoms during a decline in condition. The pathogenesis of cancer cachexia is multifactorial. It concerns insufficient oral nutrition (often caused by anorexia), increased consumption, loss of micro- and macroelements by the body, improper functioning of metabolic processes, chronic inflammation, and diametric changes caused by cancer treatment (radiotherapy or chemotherapy) [19].

One of the effects of malnutrition may be sarcopenia, which is defined as a combination of unintentional decrease in muscle mass and skeletal muscle strength and function. It is usually diagnosed in geriatric patients. The most common causes of sarcopenia, apart from the patient’s age, may be living conditions, inappropriate diet, and pharmaceuticals. One of the specific conditions is sarcopenic obesity, when, despite the loss of muscle mass, there is an increase in adipose tissue. This condition may be difficult to diagnose due to an increase in body weight or its lack of change [12].

Frailty syndrome should also be distinguished from malnutrition-related disorders. It is a mental and physical disorder that includes problems with mobility, chronic fatigue, unintentional weight loss, decreased physical activity, and intellectual disability, usually observed in people over 60 years of age [20].

It is difficult to determine the percentage of malnutrition types because of the different patient groups. According to the study by Sato et al., in patients (2,308 adults) with heart failure, 35% of patients with HF had at least marasmus malnutrition, kwashiorkor malnutrition, or both. The worst prognosis was characterized by the mixed type [21]. The study by Kadakia et al. indicates that at the time of diagnosis, 28% of patients with solid tumors are at high risk of malnutrition [22]. This shows how quickly cancer negatively affects nutritional status, which is associated with reduced food intake, lowered mood, worse mental state, and reduced appetite.

### 2.2. Nutritional Status Assessment

Screening nutritional status allows for prevention of malnutrition, which will be an element as important as its treatment. The methods of assessing the patient’s nutritional status are questionnaires: assessment scales, nutrition interview, and biochemical and anthropometric tests [23].

(1) Nutritional questionnaires concern a.o. current body weight and changes over 6 months, chronic diseases, and quantity and quality of food consumed. They are used to detect the presence of malnutrition and its severity [3,24].

(a) Nutritional Scale Risk The Score (NRS 2002) includes data on weight loss over a specified period, current BMI, and food intake, as well as correlations related to the severity of disease states. If the patient is over 70 years old, an additional point is added. The key to determining further intervention is the sum of points: if it is equal to or higher than 3, nutritional therapy should be implemented. If the sum is lower, the patient should be monitored and checked regularly. The maximum number of points is 7 [25].

(b) The subjective global scale of nutritional assessment (SGA) consists of 3 points. Point 1 is an interview concerning age, height, body weight, and gender, as well as changes in body weight, food intake, gastrointestinal symptoms, physical capacity, and nutrient requirements resulting from the disease. Point 2 is a physical examination, and point 3 is a subjective summary of the patient’s condition [26].

Despite the statutory requirement to assess the nutritional status of patients based on the NRS 2002 or SGA scale, malnutrition is still too rarely detected, or despite detection, the necessary intervention is not implemented. Other questionnaires, not used as often as the above, are the Mini Nutritional Assessment (MNA) scale and the Malnutrition Universal Screening Tool (MUST) scale [16]. A quick and easy-to-use tool is the Simplified Nutritional Appetite Questionnaire (SNAQ) scale, which is based on the assessment of appetite and allows for the limitation of the expected weight loss [7,24,27]. This also applies to the Malnutrition Screening Tool (MST) scale, which is based on short questions about weight loss (if so, specifying the amount) and food intake. The sum of the points indicates the risk of malnutrition [28].

(2) Nutrition interview, properly conducted, allows for a more precise determination of the causes and errors made by the patient. It should include information on the quantity and regularity of meals, the amount of consumption of individual product groups, the amount of fluids consumed, gastrointestinal problems, the amount of sleep, physical activity, the method of processing, and the quality of consumed products, as well as life and financial situation, in order to objectively perceive and make real changes that can be introduced in the future. Important information that should not be omitted are previously used diets, comorbidities, and medications taken [3].

(3) Anthropometric tests are a basic tool for monitoring physical parameters from the earliest years. Body mass index (BMI) is the most commonly used. It is the quotient of body mass and height (squared). Malnutrition is defined at a value below 18.5. In elderly patients, a value below 23 will indicate the risk of malnutrition [13].

Another method is to measure the skinfold above the triceps muscle and hand grip strength (HGS). Together with other anthropometric indicators (e.g., body weight, height, and BMI), it allows to complete the clinical picture of malnutrition [29,30].

Bioimpedance allows for a full analysis of body composition. Through the body composition test, it is possible to obtain precise data in which the spectrum of error is much smaller. Bioelectrical impedance is based on the difference in electrical resistance in adipose and non-fatty tissue (due to the presence of electrolytes and water) so the following results are obtained: lean body mass, amount of adipose tissue, extra- and cellular masses, and water content. Depending on the type of device, there may be more parameters obtained, e.g., calculation of the basal metabolic rate or amount of visceral adipose tissue. It is worth to mention patients who have problems with movement, where carrying out the above measurements will be significantly difficult [7].

For bedridden people, instead of height, the knee height measurement and bed scales are used so the staff does not have problems with transferring and the result is accurate. There are many anthropometric methods; therefore, there will always be an appropriate one that can be individually adjusted to the patient and their needs. This undoubtedly requires well-trained medical personnel [30].

(4) Biochemical tests are one of the basic activities of diagnosing disorders of the body’s functioning in the hospital. Due to their repeatability, they are also a good marker for detecting malnutrition but also for monitoring the effects of its treatment [23].

(a) Albumin concentration in blood is a commonly determined parameter among hospitalized patients. The decrease of albumin under the standard level is an undesirable phenomenon that may indicate the risk of malnutrition. Despite various factors influencing their amount (medical condition and hydration level), a lower value is always treated as an unfavorable factor, especially in patients referred for surgery. Due to the half-life of 18 days and the connection with cytokine activity, it is rather an indicator of chronic malnutrition. Proteins with a shorter half-life are transferrin and transthyretin (prealbumin). The latter is a protein transporting thyroid hormone, synthesized in the liver and partially catabolized by the kidneys. A serum prealbumin concentration below 10 mg/dL is associated with malnutrition. The main advantage of prealbumin compared to albumin is its shorter half-life (2–3 days), which makes it a more favorable marker of acute changes in nutritional status. Prealbumin levels may be elevated in cases of renal dysfunction, corticosteroid therapy, or dehydration, whereas they may be decreased during physiological stress, infection, liver dysfunction, and overhydration [31,32].

(b) Creatinine level reflects kidney function but also muscle mass level. CHI = (urinary creatinine excretion/creatinine excretion estimated from height) × 100 shows muscle exhaustion (deficit) [7,31].

(c) Nitrogen balance is the difference between nitrogen taken in with food and excreted (through the kidneys and skin). The values obtained are interpreted as follows: negative balance is predominance of catabolism, and positive balance is predominance of anabolism. This is influenced by the body’s protein consumption, the presence of infection or kidney function, as well as the patient’s physical condition (lying or moving) [7,30,31].

(d) As the disease intensifies and the patient’s well-being worsens, the functioning of the immune system changes, as indicated by the CLL, i.e., total lymphocyte count. The decrease under the standard level will be perceived as alarming [30].

(e) Other parameters such as liver enzymes, lipid profile, electrolytes (calcium, potassium, sodium, chloride, phosphorus, and magnesium), complete blood count, and C-reactive protein level will allow for a broader look at the patient, also in the direction of anemia and other deficiencies [7,31].

ASPEN guidelines emphasize the diagnosis of malnutrition based on the following criteria: insufficient energy intake, weight loss, muscle mass loss, subcutaneous fat loss, localized or generalized fluid accumulation, and decreased functional status through the measurement of HGS. For diagnosis, two of the six factors must be met [8].

It seems that the most optimal way to assess the nutritional status of hospitalized patients is to use the NRS 2002 scale due to its ease of use and high effectiveness. However, the problem is often not the lack of use of the tool, but the lack of awareness of the consequences that may be caused by early undetected malnutrition or its risk. Certainly, bioimpedance measurement provides a lot of valuable information, such as the level of muscle and fat tissue, which will allow for better implementation and adjustment of nutritional intervention, for example, by modifying the amount of macronutrients administered in the diet.

### 2.3. Consequences of Malnutrition

As mentioned above, if malnutrition is treated improperly or left without intervention, it will lead to death. Depending on the age of the patient, the effects will be different. Children diagnosed with malnutrition have a higher risk of mortality, along with reduced immunity to diseases caused by bacteria and viruses but also mental skills [33].

According to WHO, in 2018, growth inhibition affected 21.9%, or 149 million, children under the age of five, and 45% of deaths among children under the age of five were related to malnutrition [6]. In adults, deficiencies of micro- and macronutrients may contribute to the development of depression [7].

Malnutrition in patients after severe injuries significantly increases catabolic changes. Cytokines and hormones, such as epinephrine, glucagon, and cortisol, are released, which can increase energy expenditure by 50%. This also affects changes in protein metabolism and their conversion into energy, including muscle proteins. Consequently, if malnutrition has already occurred, it will continue to intensify. Due to the constant drawing of the body from protein stores, the ability of the body to renew cells decreases, wound healing is impaired, and changes in the immune system occur, such as the production of antibodies and the activity of white blood cells. Any loss of muscle will correlate with other effects; impairment of respiratory muscles causes a greater risk of pneumonia or respirator dependence [10]. Reduction in heart muscle mass leads to reduced exercise capacity and circulatory failure. Insufficient supply of energy and nutrients will reduce the glomerular filtration rate and affect kidney function. The effects also affect the digestive system due to the reduced absorption surface (lack of enterocyte stimulation by food); the absorption of mono- and disaccharides and fats is impaired, which leads to liver diseases [7,34].

Prevention of malnutrition should be the primary goal of medical personnel and the patient because despite appropriate treatment, it will have its consequences. Education of doctors, nurses, and other members of hospital teams and employment of dieticians allows for screening of patients at risk of malnutrition, but also for implementation of appropriate measures, such as the entire process of nutritional treatment [1]. Attention should also be paid to the nutritional awareness of patients and a certain standard of quality that should be maintained in medical facilities. Malnutrition, but also overweight and obesity, are clinical conditions that correlate with the quantity and quality of food consumed. If medical institutions and people educating patients about nutrition do not apply the established standards and provide correct nutritional recommendations, the consequences of these diseases will also be unavoidable.

## 3. Nutritional Treatment

Nutritional treatment is preceded by assessment of the patient’s nutritional status, assessment of the demand for nutrients, and determination of interventions, as well as the creation of a nutritional plan consisting of the supply of appropriate amounts of macro- and micronutrients, ongoing monitoring, and control of treatment results. The effect of a properly conducted scheme is the maintenance or improvement of the nutritional status in patients whose nutrition with a natural diet is insufficient, as well as, in some cases, treatment of the underlying disease [3]. The possibility of implementing medical nutrition is based on one of the following conditions:

- expected inability to use an oral diet for more than 7 days;
- patient’s malnutrition;
- expected inability to provide more than 60% of the recommended daily intake for a period exceeding 10 days [3].

**Figure 1:**
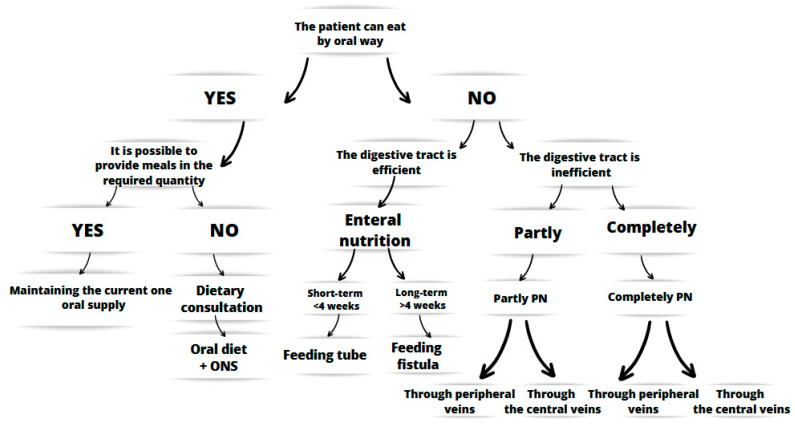
Algorithm of action in clinical nutrition [3].

The nutritional treatment of the patient is based on the following algorithm (Figure 1):

### 3.1. Oral Nutritional Supplements (ONS)

In the case where the patient does not consume adequate amounts of a natural diet, oral nutrition supplements (ONS) are used. This is one of the types of a wide range of foods for special medical purposes. There are preparations that are complete in terms of ingredients or intentionally devoid of a selected component, e.g., fat. ONS are not intended to contain ingredients such as purines, lactose, gluten, and cholesterol [35]. When consuming ONS, the time of drinking them is an important factor (at least 30 min). The division of ONS is presented in Table 1:

The wide range of products available on the market allows them to be selected for a specific disease entity. For patients with diabetes and glycemia disorders, there are products with a reduced content of monosaccharides, with a low glycemic index and containing added fiber. For people with liver diseases, low-fat or fat-free ONS can be intended, as well as with a modified fat profile and a reduced amount of protein, with the addition of branched-chain amino acid (BCAA), polyunsaturated fatty acids (PUFA), monounsaturated fatty acids (MUFA), medium-chain triglycerides (MCT), and reduced aromatic amino acids. ONS for patients with kidney diseases are usually hypercaloric, low-protein, and high-fat (mainly MCT). The amount of ingredients, such as sodium, potassium, chlorine, calcium, phosphorus, and magnesium, which could aggravate kidney problems, is reduced. In addition, there are supporting products, single-ingredient or incomplete, for example, powdered fiber, which in many cases is difficult to provide to the diet naturally, as well as thickeners for the kitchen diet [3]. Protein powder or glutamine is also available, which allows for enriching the natural diet.

Immunonutrition is characteristic for patients with neurological disorders. It is a regimen based on the supply of selected ingredients that have the ability to affect immunological and anti-inflammatory pathways [36]. Preparations of this type contain omega-3 fatty acids, glutamine, arginine, nucleotides, antioxidants, and prebiotic fiber. Omega-3 acids effectively reduce inflammation, influencing the overall improvement of treatment [37]. Components such as arginine, collagen, and fatty acids may stimulate healing and regulate the immune system response. Ascorbic acid and microelements, such as iron, zinc, and selenium or copper and individual vitamins, also support wound healing [38].

Depending on the companies offering the above preparations on the market, patients can choose ONS to suit their own preferences. This is a field of trade in a strong development phase, which allows for a wide range of effects and choices. Many products are available in liquid form, as well as individual powders to be mixed in selected solvents (milk, water, and juices). Most ONS of one type have different flavors, from fruit to traditional ones such as caramel or chocolate [39]. According to ESPEN ONS, for patients who are malnourished or at risk of malnutrition, they should contain at least 400 kcal and 30 g of protein per day. Considering the products available on the market, this is about 1–2 pieces per day, depending on the type. The use should last at least 1 month, after which the nutritional status and benefits of taking the product should be assessed [11].

### 3.2. Enteral Nutrition

The concept of enteral nutrition includes the processes of oral nutrition (which was developed above), intragastric nutrition, and enteral nutrition. Artificial nutrition through the digestive tract is used in malnourished patients who cannot provide adequate oral intake. This type of nutrition requires the proper functioning of the digestive tract, the absence of obstruction, the possibility of using artificial access, and the consent of the patient or the entity responsible for it [39].

Indications include any disorders related to:

- dysphagia, the etiology of which may be different (neurodegenerative and neurological diseases, myasthenia, and oncological diseases);
- diseases of the head and neck as well as the upper digestive tract;
- the digestive tract or its partial obstruction, also with the processes of digestion and absorption (diseases of the pancreas, intestines, liver, and allergies);
- chemotherapy and radiotherapy (cachexia or inflammatory reaction);
- chronic infections and diseases (COPD and organ failure);
- severe acute pancreatitis, loss of consciousness, extensive injuries, and procedures [39,40,41,42].

The administration of an enteral diet requires good tolerance.

Depending on the route and time of administration (over or under 14–21 days), the appropriate type of access to the gastrointestinal tract is selected for the patient’s needs. In the case of patients who are expected to receive enteral nutrition for less than 2–3 weeks, a nasogastric or nasojejunal tube is inserted. In patients who are expected to receive enteral nutrition for a longer period, permanent access to the stomach or intestine will be established, such as gatro- or jejnunostomy [39,43].

For each of the accesses inserted, when using the bolus or infusion method, it is necessary to remember about the night break. The effectiveness of selected actions should be assessed on an ongoing basis, and any modifications should be introduced [42,43,44]. Depending on the artificial nutritional access and individual tolerance of the patient, the supply of industrial diets will be different. In the case of supplying the diet to the stomach, these can be boluses and continuous infusion. Boluses are about 4–6 portions of 200–400 mL to meet the daily requirement for nutritional components. This is the target amount, the supply should start from 100 mL, and the patient’s tolerance should be monitored, such as stomach emptying and good well-being. If there are no side effects, the supply is successively increased to the target amount. In the case of supplying the diet to the intestine, only a continuous infusion is appropriate, preferably using an infusion pump. The supply starts at a rate of 15–20 mL per hour and increases with tolerance to the target value of approximately 80–120 mL per hour, which allows the diet to be administered within 16–24 h [45,46].

Industrial diets are intended for artificial access. It is dangerous and medically unjustified to administer a natural kitchen diet due to the increased risk of infection, access obstruction, and lack of appropriate energy value, i.e., exposing the patient to deepening malnutrition [45]. Depending on the patient’s condition—the disease entity, the route and method of supply, and the efficiency of the gastrointestinal tract—an appropriate diet is selected. The composition of complete industrial preparations is based on similar ingredients:

- oligo- and polysaccharides derived mainly from starch but also from beets, corn, and cane;
- long-chain triglycerides (LCT), MCT, and MUFA—vegetable oils;
- casein and whey proteins, soy protein, and egg white—in varying degrees of hydrolysis.

In patients requiring special interventions, preparations with modified amounts of macronutrients are selected [46]. Industrial diets are divided depending on the degree of protein size into mono-, oligo-, and polymeric and into those assigned to a given disease entity (similarly to ONS). Standard diets are not hydrolyzed, which is why they are characterized by the lowest osmolarity (about 300 mOsm/L). They are used in most patients, especially in the initial phase of nutrition. The content of macronutrients is similar to the proportions of the daily diet (15–20% of energy from proteins, 20–40% from fats, and the rest from carbohydrates). Examples of such diets are Fresubin Original (Fresenius Kabi, Nutrison (Nutricia) and Nutricomp Standard (Braun). In the case of modification of preparation components, the following diets are obtained: high-protein (Nutrison Protein Advance, Nutrision Protein Intense (Nutricia)), high-energy (Fresubin HP Energy (Fresenius Kabi)), and containing fiber (Fresubin Original Fibre (Fresenius Kabi)), as well as with a specific fatty acid profile (Reconvan (Fresenius Kabi)). Oligomeric preparations (containing short peptide chains) are characterized, apart from the hydrolyzed protein, also by a higher content of MCT acids, with an increased rate of digestion (Survimed OPD (Fresenius Kabi), Nutricomp Peptid (Braun), Peptamen (Nestle), and Nutrison Advanced Peptisorb (Nutricia). Their osmolarity is 300–600 mOsmol/L. The last stage of protein hydrolysis, i.e., amino acids, contains monomeric diets, which is why their osmolarity is the highest [41,44]. Hemodialysis patients receive diets with increased calories, and the percentage of protein is also increased (Fresubin Intensive (Fresenius Kabi)). In preparations for people with lung diseases, some carbohydrates are replaced with fats, which reduces carbon dioxide production and the use of the mentioned organ (Nutrison Diason Energy HP (Nutricia)). In oncological patients, as well as in perioperative care and transplantology, agents with added immunomodulating components are used (Supportan (Fresenius Kabi) and Impact Oral (Nestle)). For patients with liver failure, diets with the addition of BCAA and MCT are recommended (Fresubin Hepa (Fresenius Kabi) and Nutricomp Hepa (Braun)). Diets used in glycemic disorders and diabetes contain added fiber and reduced amounts of carbohydrates (Diben (Fresenius Kabi), Nutrison Advanced Diason (Nutricia)) [46].

### 3.3. Parenteral Nutrition

In patients whose digestive tract cannot be used or whose ability to use is insufficient, parenteral nutrition is used [47,48]. The main indications for its introduction include intestinal failure or diseases related to them, which cause the inability to absorb an appropriate amount of the enteral diet, such as short bowel syndrome, Crohn’s disease, radiation enteritis, or surgical complications [47].

Peripheral venous access is established in patients for no longer than 10–14 days or when the possibility of using central veins is excluded. Percutaneous catheters and vascular ports are used as central access. Mixtures administered to peripheral veins cannot exceed 900–1000 mOsm/L; all parenteral nutrition mixtures can be used in central veins [47].

The parenteral nutrition planning scheme includes the time of feeding, the choice of venous access, and the selection of a mixture appropriate to the patient’s needs. The mixtures are based on amino acids, glucose, and fat emulsion, and the necessary additives are water- and fat-soluble vitamins and trace elements. Immunomodulating substances such as omega-3 acids or glutamine may also be included. Depending on the type of bags, they differ in content and handling. Preparations prepared in the All-in-One (AIO) and Two-in-One (TIO) mixer contain all the ingredients and are ready for administration. Ready-to-Use (RTU) bags should be prepared for connection in the hospital pharmacy in the laminar flow cabinet, and the substances they do not contain (usually microelements and trace elements) should be injected into them. RTU mixtures may consist of two or three chambers, amino acids, and dextrose, with the addition of a fat emulsion (in the third). In terms of the content of the mixture, they will differ in the amount of nitrogen, the composition of the fat emulsion and the dextrose content, which will determine the volume of the bag, the content of macronutrients, and the energy value. Depending on the manufacturer, the composition of the fat emulsion will be different. The ingredients may be soybean oil, MCT, olive oil, and fish oil [39,47].

In patients receiving PN, the duration of the supply may last 24 h, which allows for maintaining a normal blood glucose level, but this is indicated for short-term supply, for example, during a hospital stay. In patients with home parenteral nutrition, the time of supply may be modified and with breaks in order to improve the patient’s quality of life and protect the liver. The rate of supply should depend on the clinical condition and needs of the patient, the volume of the mixture, and the stage of supply (initial or as a continuation, when the body’s reaction and tolerance are known). In long-term therapy, the time may also be shortened to 16–18 h [39,47].

It is important to monitor blood glucose levels to avoid hypo- or hyperglycemia. Slow introduction of parenteral nutrition, frequent monitoring of blood glucose levels, or the addition of insulin will help reduce excessive glucose levels. Hyperglycemia is expected in elderly patients with diabetes or multiple diseases. In the case of poorer tolerance or excessive lipid intake, hypertriglyceridemia is a common phenomenon. Risk factors include excess body weight, alcoholism, pancreatic diseases, and taking certain medications. As a preventive measure, it is recommended to add omega-3 fatty acids and reduce the supply of fats to a maximum of 1 g/kg bw/day [47].

One of the most dangerous complications is the refeeding syndrome. It is characterized by disturbances in water and electrolyte balance, especially a decrease in phosphorus levels, often also thiamine, but also the occurrence of hypomagnesemia and hypokalemia. Risk factors include low BMI, weight loss, deficiencies, reduced energy intake for more than 5 days, and diseases that intensify catabolic changes in the body. Symptoms include edema and organ failure, and without an appropriate response, they lead to death. Prevention of RS includes correcting electrolyte disturbances and deficiencies, slowly introducing nutrition starting with an energy value of 10–20 kcal/kg.bc, slowly increasing it to the target value, and continuously monitoring the patient’s condition [47,48,49].

Parenteral nutrition-associated liver disease (PNALD) is encountered. The disease combinations may include cholestasis, steatosis, and cholecystolithiasis. It may be manifested with abnormal liver parameters, steatosis, cirrhosis, or, in the worst stage, fibrosis and liver failure. Lack of adequate supply of enteral diet affects the disturbance of bile flow and gallbladder function. Factors influencing increased risk include diseases and severe clinical conditions of the patient, excessive supply of glucose and fats, and excess omega-6 acids and phytosterols derived from soybean oil. The risk is reduced by the supply of omega-3 acids, as well as not overfeeding the patient and introducing enteral nutrition in the early phase [39,47].

Prevention of complications includes continuous monitoring of the patient’s condition by checking the kidney and liver function, glycemia, and electrolyte levels at least once a week after a period of stabilization [7,47].

One of the diseases that affect high nutritional risk and increased catabolism in patients is acute pancreatitis. The older treatment regimen included the exclusion of oral nutrition in order to limit pancreatic secretion. Currently, according to ESPEN, properly conducted enteral nutrition can bring benefits, such as improvement of clinical condition and shortening of hospital stay. Parenteral nutrition should be used when enteral diet is not tolerated or there are other contraindications to enteral nutrition. It is suspected that the use of ingredients, such as glutamine and omega-3 acids, may bring benefits [7,47]. A special group is surgical patients. Nutritional preparation for procedures lasting about 7–10 days is recommended. This improves the recovery period and faster mobilization of the gastrointestinal tract. Due to the variety of procedures in patients, perioperative nutrition will also change. In patients who are expected not to be able to provide more than 50% of the recommended daily intake by the enteral route, parenteral nutrition should be implemented. Each procedure carries a risk of postoperative complications, some of which will also be indications for the inclusion of PN, for example, paralytic ileus and motility disorders. Additionally, according to ESPEN, PN supplementation with glutamine should be considered, which may shorten the hospital stay but does not affect mortality. In turn, the use of omega-3 acids reduces infections [39,47].

## 4. Inflammation

Malnutrition, among others, increases the patient’s risk of inflammation. The immune system is a protective barrier for the body against harmful factors, such as bacteria or parasites. It includes mechanisms of specific response (innate and acquired immunity) and non-specific response (e.g., physical barriers such as skin, epithelium covering the digestive tract or respiratory tract, and secretions such as lysozyme or digestive enzymes). It includes lymphatic organs and glands [50].

Inflammation can be called the body’s response to injury; it is a defense mechanism to various factors (bacterial, physical, and chemical) [51]. Acute and chronic inflammation are distinguished, depending on the duration and active mechanism. Acute inflammation is characterized by a short period of time and the initiation of PMN cells (neutrophils), secretion of inflammatory mediators by mast cells (production of histamine, other vasoactive amines, and proinflammatory cytokines), and macrophages (TNF-α, IL-1, PAF, nitric oxide, and leukotrienes), as well as components of the complement system (C3a, C4a, and C5a). The effects include vascular changes such as vasodilation, which allows the free flow of plasma with antibacterial components and antibodies. Symptoms include fever, swelling, pain, and hives. Due to neutrophils, a mechanism called oxidative burst is initiated. The key element of this process is the activation of NADPH oxidase, which catalyzes the transfer of electrons from NADPH to molecular oxygen, leading to the formation of superoxide anion (O_2_^−^). Subsequently, as a result of dismutation, this anion is transformed into hydrogen peroxide (H_2_O_2_). In the presence of the enzyme myeloperoxidase (MPO) and chloride ions, H_2_O_2_ is a substrate for the synthesis of highly reactive oxygen species, such as hypochlorous acid (HOCl) and hydroxyl radicals (•OH). The resulting compounds, due to their oxidative properties, cause damage to the structures of pathogens, including cell membranes, proteins, and nucleic acids, which ultimately leads to their elimination [52].

Once the cause of inflammation is removed, repair processes are initiated, including the secretion of anti-inflammatory and pro-inflammatory cytokine inhibitors (IL-4, IL-10, and TGF-β), as well as the secretion of collagen and growth factors by macrophages [50]. There is also the ability to activate inflammation resolution mechanisms, which counteract chronic inflammation and restore homeostasis. Pro-resolving mediators—resolvins, protectins, lipoxins, maresins, and neuroprotectins—are formed from polyunsaturated fatty acids (including EPA and DHA) and affect the modulation of the inflammatory response, inhibition of immune cell activation, and stimulation of repair processes [53].

In chronic inflammation, the inflammatory response is sustained over a prolonged period, often as a result of persistent exposure to harmful stimuli, such as pathogens, toxins, or physical injury, or due to dysregulation of the immune system. This condition is characterized by the infiltration of various immune cells, including macrophages, lymphocytes, and plasma cells, which secrete pro-inflammatory cytokines and growth factors that facilitate tissue remodeling. The prolonged activation of the immune response in chronic inflammation can result in tissue damage and play a pivotal role in the pathogenesis of several diseases, including malignancies, cardiovascular disorders, and autoimmune conditions [50].

Innate immunity cells can rely on the pattern recognition receptor (PRR) system in various forms (soluble or present on the surface and in the cell). This is related to the mechanism of pathogen-associated molecular patterns (PAMPs) and tissue damage (DAMPs). Through binding of ligand to PRRs, signaling pathways are initiated that affect the activation of transcription factor (NF-kB) and interferon regulatory factors (IRFs), which leads to the initiation of the cellular response [54]. This results in the secretion of pro-inflammatory cytokines, e.g., IL-1, IL-6, IL-10, or TNF-α and mediators (growth factors, interleukins, and chemokines) [4,54].

Cytokines are diverse proteins secreted by immune system cells that affect the regulation of the inflammatory response in a specific way, as data transmitters or directly. They can also play homeostatic roles and participate in cell formation and maturation and affect chemotaxis [55]. Several types are distinguished depending on the producing cell: monokines, lymphokines, interleukins, chemokines, and interferons [5].

Interferons, which protect against viruses by inhibiting replication—by limiting protein synthesis—are differentiated into two types: I INF (INF-α and INF-β) and II INF (INF-γ). The antiviral activity of type I interferons was confirmed in studies. After infection of animals with the virus and administration of antibodies against interferon α and β, death occurred. They also affect the reduction of cell proliferation. INF-γ participates in the activation of the immune system, affects the differentiation of lymphocytes by acting on increased cytotoxicity, and also increases the phagocytic capacity of cells in another mechanism [5].

Lymphokines include IL-2 (growth factor necessary for the proliferation of T lymphocytes), IL-3 (participation in hematopoiesis), IL-4 (growth factor necessary for the proliferation of B and Th2 lymphocytes), IL-5, IL-10, and IL-12.

Monokines are pro-inflammatory cytokines, such as IL-1, IL-6, IL-8, and IL-12 TNF-α. Their main tasks are to influence:

- increased body temperature;
- increased phagocytosis;
- secretion of acute phase proteins (CRP and MBP);
- opsonization action and complement system action.

Chemokines play a special role in the chemotaxis of selected cells of the immune system such as lymphocytes, neutrophils, and monocytes.

An important element is maintaining balance in the functioning of the immune system. Excessive levels of secreted cytokines have been observed in mental disorders. This can affect tissue damage, contributing to neurodegenerative and autoimmune diseases [5]. The influence of immunity on cancer cells is also discussed; one of the factors of their development is chronic inflammation. Individual cytokines can act in a given way. For example, IL-1α and IL-1β are involved in the secretion of carcinogenic substances, such as reactive oxygen species or nitric oxide [56]. A disease affecting a large part of society is inflammatory bowel disease. Factors influencing the occurrence of the disease include smoking, inappropriate diet, or genetic factors. Their accumulation leads to dysfunction of the intestinal barrier, which affects the movement of microorganisms and the initiation of immune cell activity. Characteristic cytokines for this process are TNF-α, IL-6, and IL-23 [57]. The action of IL-6 can be pro- or anti-inflammatory (depending on the secreted dose). It is used as a marker of inflammation in oncological patients and those burdened with autoimmune diseases due to its key role in the activation of the immune system [58]. It is an unfavorable prognostic factor in diseases of the circulatory system and pancreas [4]. It reduces the production of proteins, such as albumin, transferrin, and fibronectin, and regulates the level of zinc and iron in serum [59]. It affects liver regeneration and proper functioning, but its chronic activation can lead to the development of cancer cells [60]. TNF-α affects the secretion of acute phase proteins, especially in burns, and also has a prothrombotic effect [4,61].

Inflammation adversely affects the patient’s nutritional status. It has been shown that nutritional therapy with CRP > 100 mg/dL did not have a beneficial effect on mortality within 30 days [9]. This highlights the need for simultaneous action on treating the underlying disease and calming the inflammation, but also taking care to stop the loss of muscle mass and continuously compensate for deficiencies [9].

The intestinal microbiota performs protective, metabolic, and immunological functions. The gut-associated lymphoid tissue (GALT) system creates a selective barrier for prescribed particles of a specific size, which limits the penetration of unwanted antigens from the intestinal lumen. Factors causing intestinal dysbiosis, such as stress, a diet rich in highly processed products, drugs, antibiotics, and proton pump inhibitors, affect the deterioration of the intestinal barrier integrity, affecting the activation of GALT and inflammation [62]. As a result, pathogens enter the bloodstream. The functioning and composition of the microbiota are determined from the earliest years, beginning from colonization immediately after birth, intake of antibodies with breast milk, and protection against pathogens. It reduces susceptibility to allergy and influences the immune response in the future [63]. One of the key roles of microorganisms is the secretion of metabolites such as short-chain fatty acids (SCFA), i.e., butyric, acetic, and propionic acids, through the fermentation of fiber, affecting the integrity of the intestinal barrier. They affect the regulation of T cells and also participate in the nutrition and regeneration of colonocytes, as well as the induction of apoptosis of colon cancer cells. Butyrate produced by F. prausnitizii calms the inflammatory response by producing IL-10. Insufficient supply of fiber is the reason why other components (amino acids and fats) are used for fermentation, which leads to the formation of long-chain fatty acids. It may affect insulin resistance. Proper microbiota enables the synthesis of glutamic acid, which is one of the factors in the formation of neurotransmitters. The nervous system, sensitive to stimuli from internal organs, modulates the secretion of cortisol, dopamine, and serotonin and action of peristalsis [64]. Enterococcus bacteria affect the synthesis of IgA, inhibiting the adhesion of unwanted pathogens to the epithelium, as well as the Th1/Th2 lymphocyte balance [65]. The microbiota synthesizes selected components, such as vitamins B, K, and A. Microbiota abnormalities lead to the development of diseases, especially inflammatory bowel disease (IBD), but also cancers and metabolic disorders [66]. Selected strains can produce components with bactericidal and bacteriostatic effects [67].

## 5. Immunomodulating Ingredients

**Figure 2:**
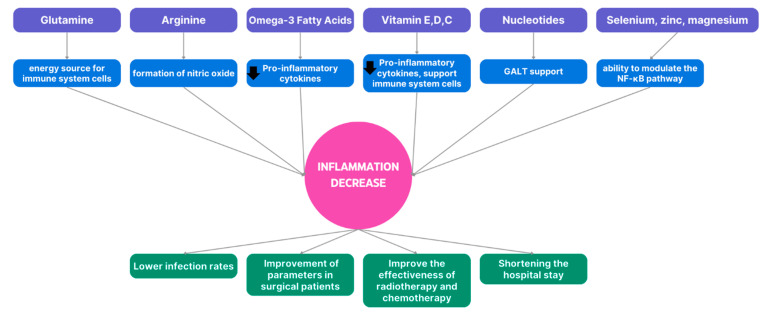
Simplified impact of immunonutrition. Immunonutrition plays a key role in modulating the immune response and reducing inflammation, which translates into improved clinical parameters for patients. Nutrients such as glutamine, arginine, omega-3 fatty acids, vitamins (E, D, and C), nucleotides, and microelements (selenium, zinc, and magnesium) have significant immunomodulatory effects, influencing the functioning of immune cells and inflammatory processes. Omega-3 fatty acids and vitamins E, D, C have the ability to reduce (as indicated by arrows) the activity of pro-inflammatory cytokines. Reducing inflammation through appropriate nutritional intervention translates into numerous clinical benefits, including reduced infection rates, improved immunological parameters in surgical patients, increased efficacy of anticancer therapies (radiotherapy and chemotherapy), and shortened hospitalization time. Consequently, immunonutrition is an important element of supportive therapy that can significantly improve treatment outcomes and quality of life of patients [68,69,70,71,72,73,74,75,76].

Many immunomodulating ingredients have an impact on improving the functioning of the immune system. This may include reducing inflammation, increasing resistance to infectious agents, increasing mucosa-associated lymphoid tissue (MALT) activity, and counteracting systemic inflammatory reaction syndrome (SIRS). In the long term, this will shorten the hospital stay and improve the nutritional status [68,69,70]. A simplified effect of several immunomodulatory substances is presented below (Figure 2):

Arginine is synthesized in the liver, kidneys, and enterocytes. It is also supplied exogenously. It participates in the synthesis of nucleotides and stimulates the activity of lymphocytes and hormones. In cachectic patients, its deficiency occurs [71,72]. This amino acid also participates in the production of citrulline and in the ornithine cycle [66]. Another process in which arginine participates is the formation of nitric oxide through the action of nitric oxide synthase. The resulting compound affects leukocyte migration, as well as granulocyte infiltration of the gastrointestinal mucosa, thereby influencing the inflammatory response [7]. This amino acid affects protein synthesis, and its deficiency impairs the proper course of the process. The dose that improves the functioning of the immune system is 3–4 times higher than the intake from conventional food, which is about 5.5 g [36]. The risk of pressure ulcers and infections, as well as inflammation, increases especially in malnourished patients. The use of oral or enteral supplementation of arinine may have a beneficial effect on both of these processes [73].

In the case of increased catabolism, the production of endogenous glutamine may be impaired and insufficient. It is a basic energy for the differentiation of intestinal epithelial cells and a source of energy for lymphocytes and macrophages; therefore, deficiency may result in ulceration of structures, intestinal villus atrophy, and intestinal barrier leakage. Exogenous supply of glutamine reduces protein breakdown and improves the process of their synthesis. Connecting the above processes improves immunity and reduces the occurrence of infections [74,75]. The surgical procedure; stress accompanying the patient; preparation for various tests, which often still involve fasting, as well as the reason for the surgery (such as obstruction or tumors in the gastrointestinal tract); and the often-complicated convalescence period are many factors that increase the risk of malnutrition. As indicated above, the use of glutamine by the enteral or parenteral route may improve parameters, such as mortality, infections, and length of hospital stay in surgical patients [38].

Omega-3 acids, i.e., eicosapentaenoic acid, docosahexaenoic acid, and alpha-linolenic acid, are exogenous compounds. They are building components of cell membranes and participate in transport processes at the cellular level, supporting the functioning of the nervous system [76]. By occurring in the cell, they may reduce the pro-inflammatory effect of some factors and improve its functioning. There are several mechanisms of action of omega-3 acids, such as inhibition of the production of pro-inflammatory eicosanoids or production of pro-resolving mediators, as well as inhibition of NF-κB activation [77]. They are characterized by antithrombotic, anti-allergic effects and have a beneficial effect on the lipid profile. They significantly reduce the level of triglycerides [77]. Omega-6 acids produce prostaglandins and leukotrienes, which are pro-inflammatory mediators. Prostaglandins can affect the occurrence of pain, redness, and swelling (by affecting sensory neurons and vasodilation-increased permeability, which facilitates immune cell migration) [78]. In turn, leukotrienes affect neutrophil chemotaxis, their interaction with the endothelium, and their activation on the release of mediators [79]. In turn, a higher supply of omega-3 acids reduces the risk of chronic inflammatory diseases and the amount of secreted pro-inflammatory cytokines [38,77,80]. A characteristic effect is also observed in cancer diseases. In oncology patients, abnormal levels of omega-6 acids have been detected, which in the presence of cyclooxygenase 2 transform into eicosanoids that affect the growth of tumors. Omega-3 acids reduce the production of prostaglandin E2, which leads to the spread of cancer [80]. They can also improve the effectiveness of radiotherapy and chemotherapy [81]. However, there is no hard evidence of a reduction in the incidence of cancer despite supplementation with omega-3 acids [82]. It has been shown that DHA is more effective in improving the lipid profile and selected inflammatory markers than EPA [76]. It may also have a beneficial effect on weight loss. The addition of EPA and DHA supplementation combined with appropriate exercise and a healthy diet significantly reduced the percentage of adipose tissue and abdominal skin folds in the study group compared to the control group [83].

Vitamin D is one of the fat-soluble vitamins. It has a multifaceted effect; its deficiency is associated with an increased risk or exacerbation of autoimmune diseases, cardiovascular system, and skeletal system. It can affect the secretion (IL-4, IL-5, and IL-10) and inhibit the secretion (IL-12, IL-6, and TNF-α) of selected cytokines. A proper level of vitamin D in the serum reduces the incidence of influenza and the severity of COVID-19 [84,85,86]; similar correlations are also sought in the treatment of neoplastic diseases, such as reducing the occurrence of micrometastases [85]. Vitamin D is involved in the process of protein synthesis, and its deficiency correlates with reduced physical performance, muscle strength, poorer postural stability, and quality of life in older people [9].

Vitamin E has a regulating effect on the immune system by strengthening the cellular and humoral response, reducing the secretion of pro-inflammatory cytokines (IL-6 and TNF-α) and changing the integrity of mucous membranes, thanks to which the function of transmitting external signals to the cell functions properly. The role of vitamin E in reducing the risk of respiratory infections, as well as some allergic diseases such as asthma, has been suggested [87,88].

Nucleotides, which participate in many biochemical processes, are components of coenzymes, triphosphates, and are the building material of nucleic acids DNA and RNA. Their influence includes beneficial effects on the gastrointestinal tract, such as the functioning of the GALT system, regeneration of intestinal villi and mucosa, and limiting bacterial translocation [68]. Nucleotide supply may also affect the maturation of T lymphocytes and the expression of IL-2, IL-6, and IL-8 [7,38]. A diet enriched with nucleotides correlates with a reduced frequency of complications and a shorter hospital stay, especially in cancer patients undergoing surgical procedures [7].

Vitamin C shortens the duration of infection and reduces the severity of symptoms, protects cells from excessive oxidation, and supports the function of lymphocytes and phagocytes [89]. It also has antithrombotic and antiviral effects and has been suggested to have a beneficial effect on the host response to the SARS-CoV-2 virus [90]. Ascorbic acid stabilizes the collagen structure, which may be beneficial in the healing process of wounds and pressure sores [39].

The role of magnesium is known in many body processes, such as DNA and RNA replication, enzyme activation, cell membrane stabilization, and muscle contraction. Magnesium also affects immune processes, such as reduced cytokine secretion by monocytes, proliferation, and lymphocyte development. In turn, magnesium deficiency has been observed to increase the level of pro-inflammatory cytokines, such as IL-6, TNF-α, IL-β, and CRP, and reduce antioxidant potential, increasing exposure to free radicals [91,92,93]. It is suspected that magnesium deficiency may affect the occurrence of diseases such as insulin resistance, type II diabetes, and cardiometabolic syndrome [93].

Selenium may have a preventive effect on cancer and neurodegenerative diseases; it is involved in processes related to the functioning of the thyroid gland and immune system. Selenium deficiency reduces the effectiveness of the immune response to infections and reduces the activity of selected cells such as T lymphocytes, macrophages, and NK cells [91]. Selenium is particularly believed to have a beneficial effect on autoimmune thyroiditis; it affects the reduction of the level of antibodies against thyroid peroxidase [94].

Zinc is a coenzyme and a component regulating the activity of enzymes; it participates in the synthesis of proteins, DNA, and RNA. The immunological activity of zinc has a multifaceted effect; among others, it supports the activity of NK cells (their correct number and activity of chemotaxis and lysis of unwanted cells) and also participates in the maturation of T lymphocytes. In the case of chronic inflammation in systemic diseases, zinc deficiency may increase the secretion of cytokines IL-β, IL-1α, and IL-6 (zinc supplementation may reduce its level in serum [95]) [91] and also affects the impairment of cellular and humoral response, reducing resistance to infections [96].

Malnutrition, in addition to protein deficiency, often also includes micronutrient and vitamin deficiencies. Patients with malabsorption syndrome, short bowel syndrome, or other conditions that can negatively affect digestion and absorption processes, such as after surgery, are particularly susceptible to deficiencies. An insufficient supply of the above-described components increases the body’s exposure to infections, contamination, and inflammation. The inflammation that already occurs affects increased protein and fat catabolism and also increases resting energy expenditure, i.e., affects increased nutrient consumption. All of these processes emphasize the inseparability of proper nutritional status and reduce the risk of malnutrition and inflammation. In turn, if one of these factors occurs, the risk of others also increases.

## 6. Research on Immunomodulatory Ingredients

In the article, Anderson and Lalla describe the possibility of using glutamine in the case of oncology patients receiving radiotherapy and chemotherapy. There is discussion about the problem of side effects of therapy, which are inflammation of the oral mucosa (mucositis) and ulcers occurring in various cases, including head and neck cancer. The following symptoms cause pain: problems with eating, which is an alarming factor for a high risk of malnutrition. There are many causes of their occurrence, direct damage to the epithelium by the applied therapy, disruption of epithelial cell division, activation of inflammatory cytokines, and tissue damage [97]. Based on the analysis of many studies, guidelines suggest the use of glutamine in oral form and/or rinsing the mouth with glutamine. This reduces the intensity of oral mucositis [97,98,99].

An interesting direction of research on the effects of L-glutamine is its role in cardiovascular diseases [100]. The study indicated oral use of glutamine as a factor improving glucose tolerance and long-term consumption correlated with a reduction in the level of systolic blood pressure and fasting glucose concentration in patients with type 2 diabetes [101,102]. Cohort studies noted a beneficial effect of glutamine consumption and an appropriate glutamine-to-glutamate ratio on reducing the risk of mortality (including that caused by cardiovascular diseases and cancer) [103].

In the study by Arribas-López et al., including an extensive review of the literature, a positive effect of glutamine supplementation on wound healing, as well as the length of hospital stay and mortality, was established [104]. A similar effect is described by Sibilska et al. in the case report of an elderly patient after an ischemic stroke with multimorbidity. After contracting COVID-19, she developed grade-4 pressure ulcers on her feet and lower leg. In addition to care, surgery efforts, dressings, rehabilitation, and the use of an anti-decubitus mattress, enteral immunomodulatory nutrition with the addition of ariginine, omega-3 fatty acids, and nucleotides was introduced. During the process, the size of pressure ulcers was measured, and photographic documentation was taken. The authors declare a reduction in wounds and progress in healing, which suggests a beneficial effect of the use of the selected immunomodulatory preparation [73].

In turn, a review based on 11 studies covering 1,079 critically ill adult patients concluded that the use of enteral glutamine did not improve mortality but only shortened the length of hospital stay [105].

In a study reviewing the data on the effect of glutamine in acute pancreatitis, 30 RCTs were included, with 915 patients in the glutamine supplementation group and 917 as the control group. Many parameters were analyzed: 13 studies included mortality, which was reduced by glutamine supplementation, especially with parenteral nutrition, compared to enteral nutrition. Five studies indicated a reduction in the patient’s stay in the ICU and, in 12 trials, a reduction in the total hospital stay (both with enteral and parenteral supplementation). Among the 646 patients examined for complications, a significant reduction in complications was observed in the study group. Individual studies also indicated an increase in serum albumin levels and, in turn, a decrease in ALT and AST levels in the case of glutamine supplementation. The values of CRP, IL-6, IL-8, and TNF-α decreased with glutamine supplementation [106].

In the guidelines for the use of immunonutrition in patients with cancer undergoing gastrointestinal surgery, the authors indicate a beneficial effect on reducing the occurrence of complications and shortening the hospital stay but do not indicate for how long such therapy should be used. In turn, in stomach cancer, oral and enteral nutrition with immunomodulating components (arginine, nucleotides, and omega-3 acids) improves immunological parameters, reduces complications, and shortens the length of hospital stay [107].

Skulas-Ray, in analysis of 43 studies on the effect of omega-3 fatty acid supplementation on inflammation (especially CRP) in various disease entities, indicates a lack of sufficient evidence to confirm the hypothesis about the anti-inflammatory effect of omega-3 fatty acids [108].

The data in the review by Nabavi et al. indicate that omega-3 fatty acids act as inhibitors of breast cancer based on several mechanisms. The first is the inhibition of prostanoid production, which regulates the inflammatory response, metastasis, apoptosis, and angiogenesis. Another mechanism involves the expression of micro-RNA—inhibiting miR-21, which reduces the growth and metastasis of breast cancer. Studies on women receiving chemotherapy with 1.8 g DHA supplementation indicated a possibility of survival of 8 months longer than without DHA supplementation, probably due to the enrichment of cell membranes with omega-3, which may increase sensitivity to chemotherapy. In the case of colorectal cancer, the effectiveness of supplementation has been proven in animal and cellular models. DHA affects the occurrence of apoptosis, inhibits proliferation in colon cancer cells in vitro, and reduces tumor formation in vivo. Omega-3 has also been shown to inhibit proliferation in gastric cancer cells, as well as improve convalescence in patients after total gastrectomy. In studies on pancreatic cancer cells that were not responding to chemotherapy, gemcitabine was given in combination with omega-3 fatty acids, which resulted in inhibition of proliferation and reduced activation of NF- κB compared to the omega-6 fatty acid control [109].

There are many studies including cell models, animal models, and human studies. A large part of them indicates a positive effect of immunonutrition, such as in patients with stomach, colon, and rectal cancer or breast cancer, or in the treatment of wounds and pressure sores. It should also be noted that not in every case the therapy will bring beneficial effects, as in patients in critical condition.

## 7. Limitations

Narrative reviews have their limitations; here, the authors point out the quantitative limitation of the review. It is not a meta-analysis. Sources were searched mainly in two browsers (Pubmed and Google Scholar), which is why some valuable papers may not be referenced. Only publications in English and Polish were taken into account; limited resources affect the lack of access to some available but paid articles. However, the review of new studies and collection of many sources of literature, as well as several discussed issues, have a positive impact on the content of the article.

## 8. Conclusions

In summary, the inflammatory state of a body may adversely affect the process of treating diseases and, among other things, due to the symptoms occurring, increase the patient’s poor well-being, lack of appetite, and low mood. A link has been shown between secreted inflammatory mediators and diseases that may worsen the patient’s nutritional status, such as cancer, inflammatory bowel disease, circulatory system, and pancreas [4,49,50]. It is crucial to highlight the special role of the patient’s proper nutritional status. The consequences of malnutrition and inflammation are well known; it is necessary to emphasize that one phenomenon will intensify the other, indicating such causes as increased metabolic demand, decreased appetite, weakening of the immune system, and intestinal dysbiosis due to deficiencies. This applies to cancer and cachexia occurring there, chronic diseases (heart failure and COPD) in which sarcopenia may occur, and intestinal diseases such as celiac disease or nonspecific inflammatory bowel diseases, which may impair the absorption of ingredients. Therefore, it is particularly important to further deepen the knowledge of the influence of immunomodulatory components in various diseases on the inflammatory state and their correlation with the nutritional status of patients. Perhaps testing specific cytokines and chemokines in selected diseases and implementing an immunomodulating component that modulates the body’s proper response will allow for in-depth knowledge of the topic and the development of further analyses. Future analyses could also consider more guidelines to detect malnutrition in primary care, before hospitalization. Develop clear guidelines on the amount and type of immunomodulatory substances in different diseases and patient groups. Consider routine supplementation of such ingredients in high-risk patients (neurological and autoimmune diseases).

## Figures and Tables

**Table 1:** Division of ONS [35].

	Preparation	Characteristic
	Hypocaloric	about 0.5–0.9 kcal/mL
Classification according to caloric value	Isocaloric	in the range of 0.9–1.2 kcal/mL
	Hypercaloric	about 1.3–2.4 kcal/mL
Classification depending on the amount of macronutrients	High energy High protein	fat content increased by about 30% protein content increased by at least 20%
	Mixed	-
	Polymeric	polypeptides
Classification depending on the degree of protein hydrolysis	Oligomeric and monomeric	oligopeptides and amino acids

## Data Availability

Not applicable.
